# Hybrid Systems Based on Talc and Chitosan for Controlled Drug Release

**DOI:** 10.3390/ma12213634

**Published:** 2019-11-05

**Authors:** Luciano C. B. Lima, Caio C. Coelho, Fabrícia C. Silva, Andréia B. Meneguin, Hernane S. Barud, Roosevelt D. S. Bezerra, Cesar Viseras, Josy A. Osajima, Edson C. Silva-Filho

**Affiliations:** 1 LIMAV, Interdisciplinary Laboratory for Advanced Materials, Federal University of Píaui, Campus Universitário Ministro Petrônio Portella, Teresina, Piaui 64049-550, Brazil; brandao_lc@hotmail.com (L.C.B.L.); caiocarvalhocoelho1@gmail.com (C.C.C.); josy_osajima@yahoo.com.br (J.A.O.); 2 Campus Senador Helvídio Nunes Barros—UFPI/CHHNB, Picos, Píaui 64607-607, Brazil; briciaquimica@hotmail.com; 3 Research Center on Biotechnology—Uniara, Araraquara, São Paulo 14801-340, Brazil; abagliottim@hotmail.com; 4 Biopolymers and Biomaterials Laboratory (BIOPOLMat)—Uniara, Araraquara, São Paulo 14801-340, Brazil; hernane.barud@gmail.com; 5 Federal Institute of Piauí, Teresina-Central Campus, IFPI, Teresina 64000-040, PI, Brazil; rooseveltdsb@ifpi.edu.br; 6 Department of Pharmacy and Pharmaceutical Technology, University of Granada, 18071 Granada, Spain; cviseras@ugr.es

**Keywords:** phyllosilicate, chitosan, hybrid, amiloride, drug release

## Abstract

Inorganic matrices and biopolymers have been widely used in pharmaceutical fields. They show properties such as biocompatibility, incorporation capacity, and controlled drug release, which can become more attractive if they are combined to form hybrid materials. This work proposes the synthesis of new drug delivery systems (DDS) based on magnesium phyllosilicate (Talc) obtained by the sol–gel route method, the biopolymer chitosan (Ch), and the inorganic-organic hybrid formed between this matrix (Talc + Ch), obtained using glutaraldehyde as a crosslink agent, and to study their incorporation/release capacity of amiloride as a model drug. The systems were characterized by X-ray diffraction (XRD), Therma analysis TG/DTG, and Fourier-transform infrared spectroscopy (FTIR) that supported the DDS’s formation. The hybrid showed a better drug incorporation capacity compared to the precursors, with a loading of 55.74, 49.53, and 4.71 mg g^−1^ for Talc + Ch, Talc, and Ch, respectively. The release assays were performed on a Hanson Research SR-8 Plus dissolver using apparatus I (basket), set to guarantee the sink conditions. The in vitro release tests showed a prolongation of the release rates of this drug for at least 4 h. This result proposes that the systems implies the slow and gradual release of the active substance, favoring the maintenance of the plasma concentration within a therapeutic window.

## 1. Introduction

The combination of materials comes from the need for better performance and properties in order to improve those observed in the precursor materials, aiming for application optimization and innovation. When two materials of different natures, organic and inorganic, are combined at the molecular level, a new material is created, a hybrid with new features, called inorganic-organic hybrids [1,2,3,4].

In the pharmaceutical area, organic-inorganic hybrid materials are investigated to improve existing properties of starting materials, aiming to have a better interaction with the drug and promote its controlled release in the organism [5,6,7,8,9]. 

Different materials for controlled release are reported in the literature, which have in common the use of phyllosilicates, which is justified because these materials are abundant in nature, are low cost, non-toxic, and have the ability to adsorb ions and/or molecules in their interlamellar space [10,11]. In addition, there is the potential diversity of their use, which is due to the ease with which these materials are modified [12,13,14,15].

Phyllosilicates may interact with chitosan, giving rise to a new mineral-polymer matrix [16,17,18,19]. Chitosan is a natural copolymer, obtained by the deacetylation of chitin, with good properties of biocompatibility, antimicrobial activity, biodegradability, and reduced toxicity [20]. These characteristics contribute to growth and exploitation in the formulation of drug delivery systems [21,22].

These matrices are a type of material that is studied extensively for drug delivery systems due to the practicality and low cost [16,21,22,23,24]. These can control release by dispersing the drug contained within or adsorbed to the surface of a porous polymer or mineral matrix [22,25,26].

In controlled release, the administered doses of drugs can be kept in an effective concentration range longer with less variation, due to the maintenance of therapeutic levels, minimizing possible undesirable effects, and improving the efficiency of treatment [27,28,29].

In conventional clinical methods, drugs can cause various collateral effects due to the large amounts of the drug administered, which can lead to patient intoxication. In addition, the drug is released in an increasing rate that, after some time, decreases rapidly requiring a new dose [24,30,31], as occurs with many medications.

**Figure 1:**
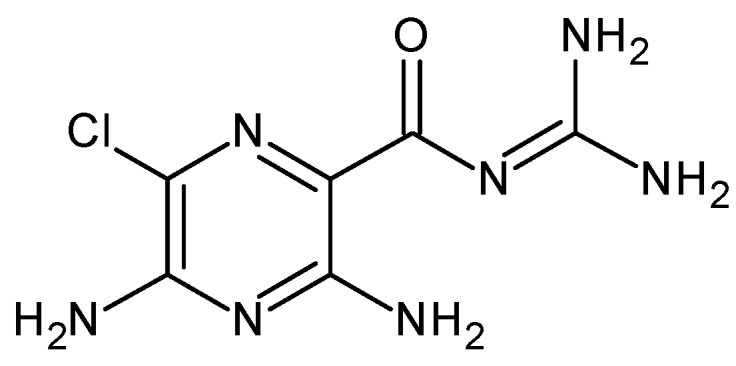
Chemical structure of amiloride.

The chemical structure of amiloride (3,5-diamino-6-chloro-*N*-(diaminomethylene)pyrazine-2-carboxamide) is presented in Figure 1. It has a pKa of 8.7, LogP = −0.3, solubility = 1.22 g, and was used in this work as a model drug. It is an oral diuretic drug that promotes the retention of potassium by the organism and the reduction of sodium and chlorine by excretion and hypertension. It is often used as a booster for other diuretics and to combat congestive heart failure as it has a weaker action compared to thiazide diuretics [32,33,34,35].

Reasonable dosages of diuretics may avoid collateral effects [36], therefore, the formulation of a controlled release system for these drugs is of great value, as most of these medicines already have a similar behavior in the organism, characterized by an increase and subsequent reduction of concentration after its administration [33].

Based on this, this work proposes the synthesis of new drug release systems based on magnesium phyllosilicate (Talc) obtained by the sol–gel method, the chitosan biopolymer (Ch), and the inorganic-organic hybrid formed between these matrices (Talc + Ch) in order to evaluate its incorporation/release capabilities of amiloride as a model drug.

## 2. Results and Discussion

### 2.1. Characterizations

**Figure 2:**
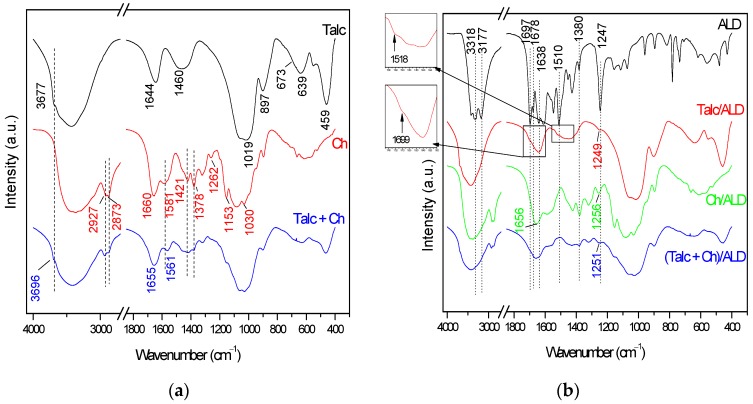
Fourier transform infrared (FTIR) spectra of (**a**) talc-like phyllosilicate (Talc), chitosan (Ch), and the hybrid based on talc-like phyllosilicate and chitosan (Talc + Ch). (**b**) Drug delivery systems obtained after adsorption of amiloride (Talc/ALD; Ch/ALD and (Talc + Ch)/ALD).

Figure 2 shows the Fourier-transform infrared (FTIR) spectra for amiloride and its interactions with each material after adsorption. 

For synthesized talc (Talc in Figure 2a), the region above 3000 cm^−1^ represents the contribution of the bands that correspond to the Mg–OH stretch vibration in 3677 cm^−1^, as well as the –OH stretch present in the silanol groups and in adsorbed water molecules around 3434 cm^−1^. The band in 1644 cm^−1^ represents the OH deformation of adsorbed water molecules. The tetrahedral layer of the phyllosilicate is represented by the bands in 1019, 897, and 459 cm^−1^, which are attributed to asymmetric and symmetrical stretch vibration modes, Si–O–Si deformations, respectively. Meanwhile, the octahedral sheet is indicated by the bands in 673 and 639 cm^−1^, associated with the deformations Mg–O and Mg–O–Si, respectively [37], just like the band in 551 cm^−1^ corresponds to stretch vibration Mg–O [38,39]. The broad band around 1460 cm^−1^ and the shoulder in 1063 cm^−1^ can be attributed to δ(CH_2_) and Si–O–C vibration, respectively, which indicate the presence of remaining ethoxy groups from the incomplete hydrolysis of tetraethyl orthosilicate (TEOS).

In the Ch spectrum shown in Figure 2a, the region from 3800 to 2700 cm^−1^ has the contribution of the absorptions referring to the OH and N–H stretching vibrations, which overlap forming the asymmetric broad band above of 3000 cm^−1^, as well as the bands in the region between 2927 and 2873 cm^−1^ attributed to the C–H stretch. The bands between 1700 and 1200 cm^−1^ represent the characteristic absorptions of the presence of amide groups C=O in 1660 cm^−1^ [40]. The contribution of OH and NH_2_ group deformations can be noticed in the band at about 1581 cm^−1^, and the axial amide deformation –CN and angular deformation of CH_3_ are seen in 1421 and 1378 cm^−1^, respectively. The characteristic band of the presence of amide II is observed in 1262 cm^−1^ [41,42]. The region with absorption between 1200 to 800 cm^−1^ is attributed to the chitosan saccharide structure, with absorption in 1153 and 1030 cm^−1^, attributed to the C–O–C stretch of the glucopyranoside ring and the β-1,4 bond [40,43,44,45].

Hybrid formation should occur through interactions of OH groups on the Talc surface with OH and/or NH_2_ groups on Ch, through glutaraldehyde cross-linking. Since these groups may be overlapping in the same spectral region, the clear attribution of their interaction is difficult. Even so, some differences in the Talc + Ch spectrum in Figure 2a indicate the effectiveness of synthesis. First, the broad band spectrum difference above of 2700 cm^−1^, as well as the displacement of the band from 3677 to 3696 cm^−1^, indicates the interaction between OH and NH_2_ groups by GLA cross-linking and/or the formation of differentiated hydrogen bonds [22]. The displacement of the band from 1581 cm^−1^ of Ch to 1561 cm^−1^ is also indicative of crosslinking from the amino group [23,46]. The band present in the region between 1200–900 cm^−1^ shows the overlap of the characteristic bands of the Talc and Ch precursors, as well as the new Si–O–C and C–NH–C vibrations, acquired from GLA crosslinking, which changed the appearance of the band compared to the precursors. 

The graphs shown in Figure 2b show the spectrum of amiloride (ALD) and materials after drug incorporation. In the spectrum of amiloride it is possible to identify its main bands in the regions of 3318 and 3177 cm^−1^, responsible for stretching the N–H bond. The bands in 1697 and 1678 cm^−1^ are associated with the double bond stretch C=O. In the region between 1550–1350 cm^−1^ are the bands relative to the C=C and C=N bonds of the aromatic ring, as well as the characteristic signs of the NH groups. Finally, at 1247 cm^−1^ is the sign for N–(C_6_H_6_) stretch [47,48].

In the Talc/ALD spectrum (Figure 2b), incorporation is indicated by the appearance of an absorption band in 1249 cm^−1^ relating to stretching N–(C_6_H_6_) and shoulders in the regions between 1720 to 1685 cm^−1^ and between 1550 to 1400 cm^−1^ that can be attributed to the interaction between the hydroxyls present on the Talc surface with carbonyl (C=O) and the ALD amino groups, respectively, forming hydrogen bonds.

When compared to the Ch spectrum, Ch/ALD has a band at 1256 cm^−1^ intermediate to the 1247 cm^−1^ observed for ALD and 1262 cm^−1^ for Ch, which indicates an interaction between the drug N–(C_4_N_2_) stretch and the amide II of the chitosan, beyond the displacement of the band from 1660 to 1656 cm^−1^ due to the group interaction of –OH and/or C=O of the amides in Ch with the carbonyl group of ALD by hydrogen bonding [49], confirming the incorporation of the drug.

Regarding the incorporation of ALD into the Talc + Ch hybrid, an enlargement in the band in 1561 cm^−1^ can be observed, which is attributed to the contribution of amiloride modes due to the interactions between amino groups (–NH_2_) of Ch and Si–OH of the Talc surface [50]. A similar effect is observed in the band at 1655 cm^−1^, attributed to the interaction of the –OH group of Talc and Ch with the –NH group of ALD by hydrogen bonding [49]. In addition, a band appears in 1251 cm^−1^, which is very similar to the absorption of 1247 cm^−1^ in the ALD spectrum, which occurs as well in Talc/ALD and Ch/ALD, indicating the interaction between N–(C_4_N_2_) and the materials.

**Figure 3:**
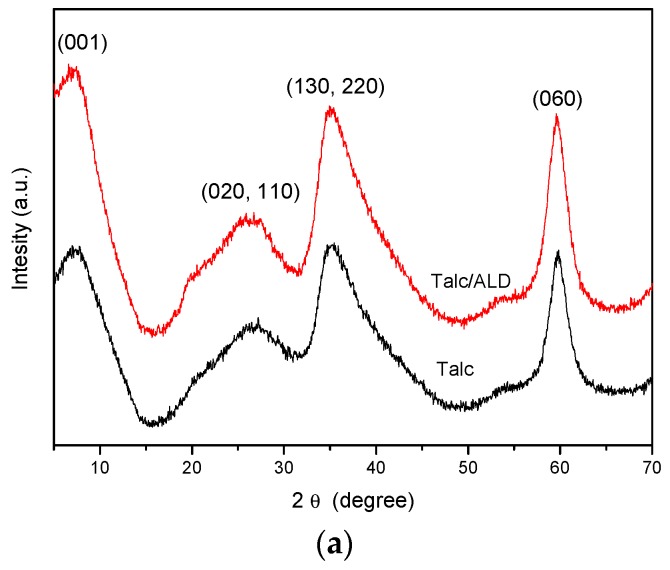

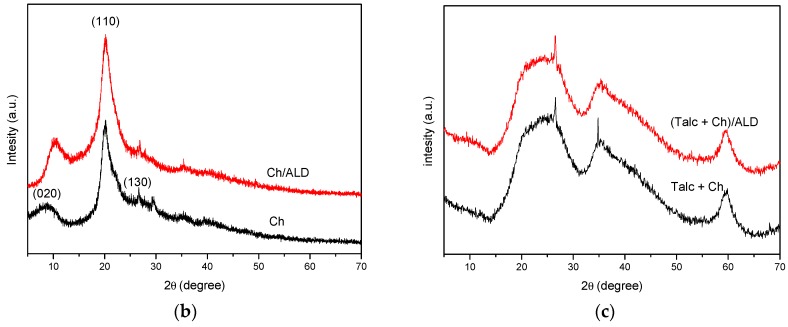
X-ray diffractogram (XRD) patterns of powders obtained before and after adsorption of amiloride (ALD) on (**a**) talc-like phyllosilicate (Talc), (**b**) chitosan (Ch), and (**c**) hybrid composite based on talc-like phyllosilicate and chitosan (Talc + Ch).

The results obtained from X-ray diffraction (XRD) are presented in Figure 3. In Figure 3a, it can be seen that the diffractogram representing the synthesized inorganic matrix (Talc) shows broad peaks defined at 7.2°, 26.3°, 35.1°, and 59.5°, identified as reflections in the crystalline planes (001), (020, 110), (130, 220), and (060), respectively. The peak representing the basal plane (001) corresponds to an interlamellar distance *d_001_* equal to 1.23 nm. The reported lamella thickness for natural Talc is about 0.96 nm [39], thus, the interlamellar spacing obtained is approximately 0.27 nm. The maximum length estimated by the Chem Sketch 12.0 software (ACD/Labs, Toronto, ON, Canada) for ethoxy groups is about 0.16 nm, indicating that the remaining groups of incomplete TEOS hydrolysis may be within the basal space and/or on the Talc surface. The reflection related to the plan (060), observed at 2θ = 59.5°, corresponds to trioctahedral characteristics of type 2:1 phyllosilicates as in natural talc [51]. The presence of these characteristic peaks proves the efficiency of synthesis, indicating that the synthesized structure resembles the structure of natural Talc [37,52,53].

The diffraction pattern of the materials was maintained after ALD adsorption, showing no structural changes with drug incorporation. Even so, minor changes can be observed. In the case of the Talc/ALD system (Figure 3a), the peak for basal spacing is not displaced, indicating that incorporation should preferentially occur on the surface of talc synthesized with the possibility of partial intercalation of the ALD molecule. The interlamellar spacing of 0.25 nm (obtained after subtraction of the lamella thickness from 0.96 nm) can accommodate both the aliphatic portion, with nitrogen groups forming hydrogen bonds in the basal space, leaving the aromatic portion interleaved, as well as the opposite, wherein the aromatic ring is perpendicular to the lamella. In addition, it is possible to observe the elevation of a shoulder at characteristic 20.5° of organic-mineral compounds [52] and a slight increase in the amorphous region of the material between 45° and 55°, which indicates drug intercalation. 

Through the chitosan diffractogram in Figure 3b, it is possible to observe a peak around 8.7° of plane deflection (020), a peak at 20.1° from the plane (110) and another of low intensity at 35.4° referring to the crystalline region of chitosan, due to hydrogen bonds between amino and hydroxyl groups in Ch chains. The peak at 26.7° of plane deflection (130) is said to correspond to acetylation in the form of acetates and free base [40,54,55].

Comparing the diffractograms in Figure 3b, it can be observed that the peak at 8.7° displaced to 10.3° with increased intensity and narrowing, which can be attributed to interaction with ALD [54]. An increase in peak intensity by 20.1° (110) was also observed.

The diffractogram of the synthesized Talc + Ch hybrid can be seen in Figure 3c. In this new crystallographic profile, the characteristic peaks of each of the original components and/or combinations of these can be noted. In the formation of the hybrid, biopolymer cross-linking to the Talc surface occurs through crosslinks promoted by glutaraldehyde. The coating of the phyllosilicate by the semicrystalline polymeric chain of Ch promotes a disorder in the periodicity of the planes (001), which implies the disappearance of the peak below 2θ = 10°. The broad peak between 17° and 29° is associated with the contribution of Ch and Talc, indicating the cross-linking of chitosan to phyllosilicate. The presence of peaks at 35.1° and 59.3° are acquired characteristics of the structure of magnesium phyllosilicate, besides presenting a larger number of amorphous domains than in Talc and Ch. The diffractogram representing the organic-inorganic hybrid after amiloride adsorption (Figure 3c) does not show significant differences, maintaining the structural profile of the hybrid.

**Figure 4:**
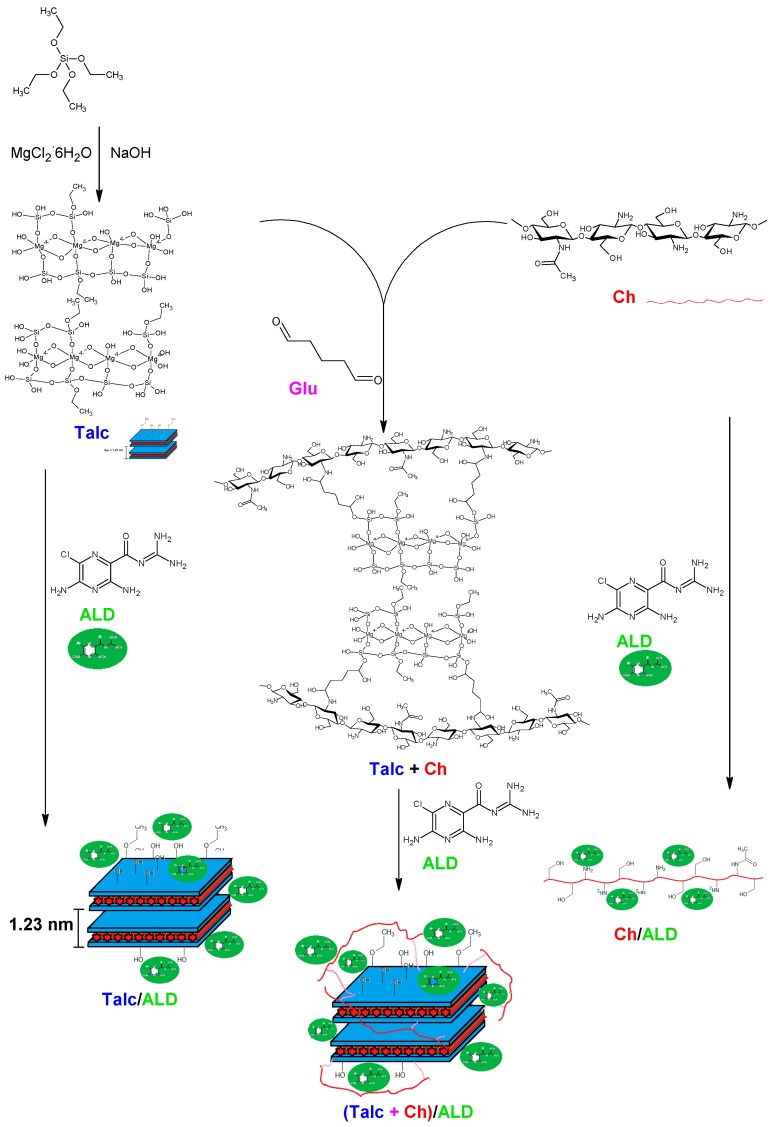
The purpose mechanism for the production of materials and the loading of ALD.

With the characterizations were proposed structures for the studied materials, which are presented in Figure 4. Incorporation of the drug into the systems (Talc/ALD, Ch/ALD, and (Talc + Ch)/ALD) is promoted by hydrogen bonds between the amino groups and/or π bonds present in the aromatic ring of ALD, which interact with groups on the surface of materials. In the specific case of incorporation of ALD into the hybrid (Talc + Ch)/ALD, there is a possibility of trapping ALD molecules in the space between the Talc surface and the polymeric chains of Ch that form cross-links by the GLA.

**Figure 5:**
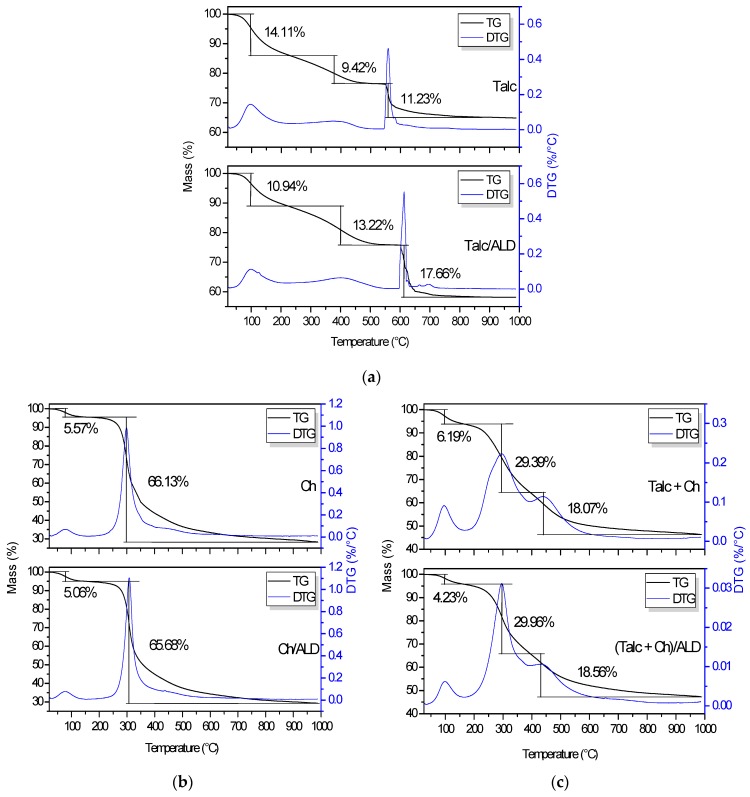
Thermogravimetric (TG) and corresponding derivative TG (DTG) curves of samples obtained before and after adsorption of amiloride (ALD) on (**a**) talc-like phyllosilicate (Talc), (**b**) chitosan (Ch), and (**c**) hybrid composite based on talc-like phyllosilicate and chitosan (Talc + Ch).

The TG/DTG curves of the samples are shown in Figure 5. The TG curve representing magnesium phyllosilicate (Figure 5a) shows three thermal degradation events. The first event occurs between 28 and 231 °C, with a mass loss of 14.11% attributed to water loss physically linked to the lamellar surface (max. 96.7 °C). The second event appears in the region between 231 and 514 °C (max. 377.8 °C) with a mass loss of 9.42%, where loss of organic matter is generally reported in thermal analysis of clay minerals, which can be attributed to the loss of the remaining O–C_2_H_5_ groups from the synthesis. In the third event, there is a mass reduction of 11.23% between 543.9 and 841.4 °C, which is related to collapse of the phyllosilicate lamellas due to the removal of hydroxyl groups from the –Si, –OH, and –Mg–OH structures that form water, resulting in siloxane condensation [37,52,56]. The observed residual mass was 65.24%.

In the Talc/ALD system (Figure 5a), the physisorbed water loss is noted in the range of 27.6 to 221.4 °C, corresponding to the DTG maximum peak at 98.8 °C and equivalent to the mass loss about 10.94%. When compared to Talc, the decrease in the percentage of mass loss and the displacement of the maximum temperature of this event are associated to the incorporation of ALD. The increase in the percentage of lost mass from 9.42% to 13.22% in the second thermal event, where organic matter decomposition begins, is evidence of the presence of ALD. In the third event, the mass loss of 17.66% from 596.2 °C is similar to what happened with Talc, by the collapse of the lamellas and removal of hydroxyls in –Si–OH and –Mg–OH, with the contribution of the decomposition of sub-products of the ALD. An increase in thermal stability compared to Talc may also be observed. this behavior is evident in the second event (max. 398.9 °C) and third event (max. 596.2 °C) which shifted at about 50 °C of difference compared to the same event in Talc. It is probably due to the action of the sub-products of ALD decomposition, which difficult the loss of hydroxyl groups from the –Si–OH and –Mg–OH structures [57]. The residual mass less than in Talc, 58.15%, is probably attributed to the formation of chemical species of higher volatility, resulting from the interaction between Talc and ALD at high temperatures.

TG/DTG curves for purified chitosan are presented in Figure 5b and show two thermal degradation events. The first event was due to water loss of surface humidity, occurring between 28.10 and 164.70 °C and adding 5.57% mass loss (max. 78.9 °C). The second has a mass loss of 66.13%, starting at 164.7 °C and extending to the end of the curve at 987.7 °C, with a maximum loss at 299.0 °C associated with the thermal degradation of the polymer [22,58]. The observed residual mass was 28.30%.

In the Ch/ALD system, present in Figure 5b, water loss between 25.6 and 152.0 °C was observed, totaling a 5.06% mass loss (max. 76.5 °C). In the second thermal event, there was a loss percentage of 65.68% of mass between 152.0 °C and 987.5 °C (max. 307.9 °C), a degradation temperature slightly elevated compared to pure chitosan, due to the presence of adsorbed ALD. In this range, a small signal at 436.7 °C, concerning the sub-products of thermal decomposition of ALD, was also observed by DTG. Another characteristic of the presence of ALD is the higher elevation of the derivative peak at 307.9 °C in relation to pure Ch. The observed residual mass was 29.27%, higher than that observed in Ch, due to the presence of residual ALD.

TG curves for the Talc + Ch hybrid before and after ALD incorporation are presented in Figure 5c and show three events. The first show the loss of physisorbed water between 34.6 and 166.8 °C, adding a 6.19% loss (max. 96.1 °C). The second event shows 29.39% mass loss between 166.1 and 394.8 °C, with a maximum at 295.0 °C. The loss percentage of this event is intermediate between the behaviors already observed for Talc and Ch in the same temperature range, representing the degradation of chitosan and the contribution of organic matter from the Talc structure, indicating the crosslinking of the hybrid. In the third event, the mass loss about 18% occurred from 394.8 °C to 800.2 °C (max. 439.1 °C). This event can be assigned to the collapse of the phyllosilicate lamellas due to the removal of the structural hydroxyl groups in –Si–OH and –Mg–OH, carbon chain degradation of GLA and remaining organic matter. The observed residual mass was 46.35%.

Finally, in the (Talc + Ch)/ALD system three events were also observed. The first is related to water loss (4.23%) between 34.1 and 164.4 °C (max. 98.6 °C). The second corresponds to 29.96% of the mass and occurs between 164.4 and 394.4 °C, with a maximum at 295.8 °C, which, besides the characteristic loss of the hybrid formed, also refers to the degradation of the adsorbed ALD. The third thermal event showed a loss of 18.56% from 394.4 °C to 874.7 °C (max. 430.1 °C), equivalent to Talc + Ch isolated. The observed residual mass was 47.31%, higher than the residue in the pure hybrid, as with Ch/ALD.

The synthesis was performed in triplicate, the characterizations of three materials were obtained, and no differences were observed in any of the characterizations used.

### 2.2. Drug Loading Efficiency and Release Tests

Through Equation (3) it was possible to determine the adsorbed amount of amiloride in each material, as shown in Table 1.

The results presented in Table 1 corroborate with the hypothesis that the formed hybrid has active sites for drug adsorption similar to the amount of phyllosilicate sites, since the amounts adsorbed by both were close. When compared to chitosan, there is a significant increase in the amount adsorbed.

**Figure 6:**
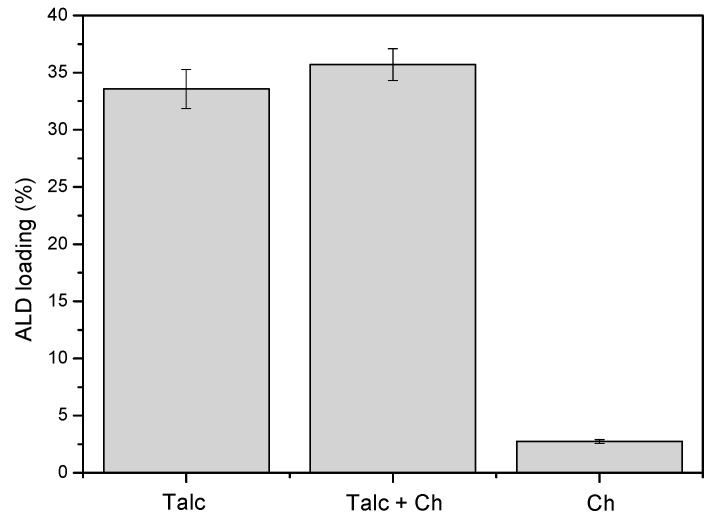
Comparison of the efficiency of incorporation of ALD in Talc, Ch, and Talc + Ch.

In terms of percentage of adsorbed drug (Figure 6), it is observed the following order: Talc + Ch hybrid (35.71%) > Talc (33.56%) > Ch (2.74%). The smaller incorporation capacity for Ch can be attributed to the low interaction between the drug amine and chitosan groups. The small increase in the amount of drug adsorbed to the hybrid may be due to an increase in hydroxyl functions, present in Talc and Ch with the crosslinking, which improve adsorption efficiency [22,59,60], as well as the possibility of incorporation into the space formed between the Talc surface and the Ch chains that cover the hybrid.

Thus, Talc and Talc + Ch are the most interesting materials in this work for use in the adsorption of ALD, since these adsorbed considerable amounts in relation the administration concentrations for this drug [61,62]. While the chitosan presented a low drug adsorption potential.

**Figure 7:**
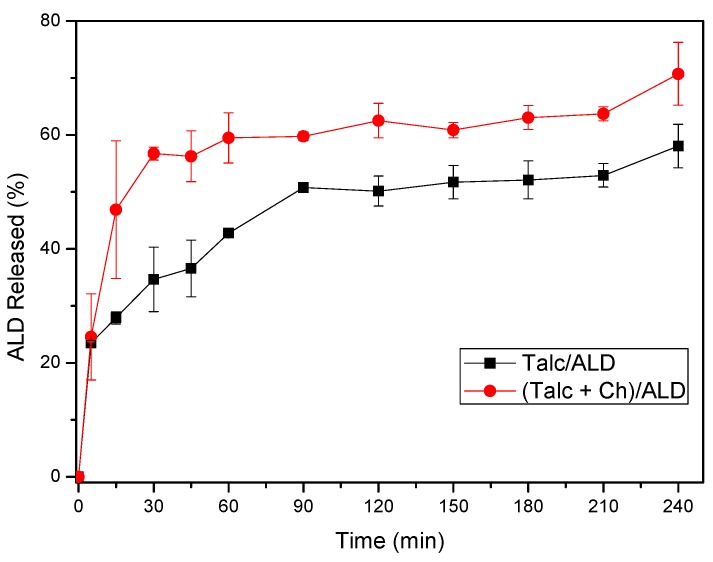
ALD release profiles from samples Talc/ALD and (Talc + Ch)/ALD.

The evaluation of in vitro release of ALD from the developed systems showed the prolongation of the release rates of this drug for at least 4 h, as observed in Figure 7. This prolongation of release proposes that the materials developed can be applied as sustained drug release system, which implies the slow and gradual release of the active drug, favoring the maintenance of plasma concentration within a therapeutic window. These systems have the advantage of providing a lower frequency of drug administrations, associated with reduced incidence of collateral effects and, consequently, a greater patient compliance with treatment [63].

For the different samples evaluated, the release rate of ALD from Talc/ALD samples, compared to (Talc + Ch)/ALD samples, offered control for the amount of drug released, with a 17% reduction in drug released after 240 min of testing. In this study, the Ch/ALD material did not present sufficient amounts of the incorporated drug that could enable the release evaluation.

An immediate release effect of approximately 56% was observed for the (Talc + Ch)/ALD sample within the first 30 min, which is related to the desorption of molecules that have low interaction with the hybrid, i.e., those that interact with the Talc + Ch surface. Subsequently, the rates remained practically constant, where the gradual slow release may be associated with ALD desorption within the space between the chains of Ch that form cross-links with the Talc surface, being that at the end of the test, approximately 70% of ALD was released.

The Talc/ALD sample showed a gradual increase in release rates up to 90 min into the test, with 51% desorbed. This is due to the desorption of the ALD molecules that are incorporated on the surface of the inorganic matrix [64]. Then, the release remained practically constant until the end of the test, with only 58% ALD released, showing a better retention of ALD molecules probably due to the partially intercalation in the interlamellar space, suggesting a strong interaction between ALD and Talc.

Results, based on ANOVA and t-test, confirm that there is a significant difference in the drug release of the two formulations after 15 min.

The mathematical models of Baker–Lonsdale, Higuchi, Korsmeyer–Peppas, First-Order, Hixson Crowell, and Weibull were applied to the release data (Figure 7) and the adjusted correlation coefficients (r^2^) were determined. The coefficient values for the various mathematical models are presented in Table 2.

From the correlation coefficient values, the mathematical models that best correlated with the release data of the studied systems were Korsmeyer–Peppas and Weibull.

The mathematical model of Weibull exponentially relates the fraction of the drug released at the time *M_t_* by the total amount released in infinite time *M_∞_*, with *a* and *b* being the scale and shape parameters, respectively. The equation for the Weibull model is shown in Equation (1) and must be applied to the data of the first 63.2% of release of the drug [65].

(1) $$\frac{M_{t}}{M_{\infty }}=1-exp\left(-at^{b}\right)$$

The value *b* is an indicator of the drug transport mechanism, where for values of *b* ≤ 0.75, the release occurs through Fickian diffusion (governed by Fick’s Law). While 0.75 < *b* < 1 has Fickian diffusion associated with Case-II and for *b* > 1, there is a complex release mechanism [66].

Already, the Korsmeyer–Peppas model is based on the Law of Powers and exponentially correlates drug release with time and fraction of drug released, $M_{t}/M_{\infty }$, with *K*, a kinetic constant, and *n*, the liberating exponent. Equation (2) represents the model.

(2) $$\frac{M_{t}}{M_{\infty }}=Kt^{n}$$

According to exponent *n*, the release mechanism can be characterized as follows:

- *n* = 0.43: Fickian diffusion (Case-I);
- 0.43 < *n* < 0.85: Non-Fickian transport (anomalous);
- *n* = 0.85: Case II transport;
- *n* > 0.85: Super-Case II transport.

For the (Talc + Ch)/ALD sample, the model that best correlated with ALD release data was Weibull’s (*r*^2^ = 0.9830). As the value of *b* was less than 0.75 (*b* = 0.1809), it is concluded that the release occurred through Fickian diffusion, according to a concentration gradient, i.e., the drug is moved to the medium of lower concentration of ALD.

Although the Talc/ALD sample correlated better with the Korsmeyer–Peppas mathematical model (*r*^2^ = 0.9821), this model is only applicable to polymeric systems [67]. In this case, it must be assumed that the release of the ALD was governed by the Weibull model (*r*^2^ = 0.9390), with a *b* value < 0.75, also characterizing the release as Fickian diffusion.

## 3. Materials and Methods

### 3.1. Materials

Tetraethyl orthosilicate (TEOS) 98% (Sigma-Aldrich, São Paulo, SP, Brazil); magnesium chloride hexahydrate 99% (Impex, São Paulo, SP, Brazil); sodium hydroxide 99% (Vetec, Rio de Janeiro, RJ, Brazil); ethyl alcohol 99.5% (ISOFAR, Duque de Caxias, RJ, Brazil); ethyl alcohol 70% (Impex, São Paulo, SP, Brazil); sodium hypochlorite (4–6%) (Vetec, Rio de Janeiro, RJ, Brazil); acetic acid glacial 99.7% (Chemco, Hortolândia, SP, Brazil); Glutaraldehyde (GLA) 50% (Impex, São Paulo, SP, Brazil), amiloride hydrochloride (ALD) (Fagron, São Paulo, SP, Brazil), and chitosan (Ch) (deacetylation degree 78%) (Polymar, Fortaleza, CE, Brazil). 

### 3.2. Synthesis of Magnesium Phyllosilicate (Talc)

Magnesium phyllosilicate was prepared following the previously reported methodology [51,68,69] with adaptations. It had a molar ratio of 4/3 (Si/Mg), which is analogous to the stoichiometric relationship found in natural Talc. In the synthesis, the amount of the tetraethylorthosilicate silylating agent as source of silicon in 40.0 mmol (9.38 mL) was calculated considering the mass of Si and the amount of magnesium chloride as a source of magnesium in 30.0 mmol (6.50 g), considering the mass of Mg. TEOS was dissolved in 75.0 mL of ethyl alcohol and the magnesium chloride dissolved in 75.0 mL of distilled water, in order to satisfy the stoichiometry according to their molecular weight.

The TEOS solution was dripped onto the magnesium chloride solution already contained in a beaker under magnetic agitation and without temperature control. After the addition was complete, sodium hydroxide (0.50 mol∙mL^−1^) solution, in the pre-calculated volume, was rapidly added. The system was kept under agitation for 2 h more for base incorporation and then aged for 7 days to ensure phyllosilicate condensation. After this period the alcohol was washed and the solids were then oven dried at 60 °C for 24 h. Magnesium phyllosilicate, named Talc, was macerated and stored.

### 3.3. Purification of Ch

Purification followed the previously described method [22]. A suspension of Ch in distilled water was prepared and 3.6% sodium hypochlorite was added under magnetic agitation for 1 h. Subsequently, the same was centrifuged at 3000 rpm for 5 min, washed with distilled water, and oven dried at 50 °C for 3 days.

### 3.4. Synthesis of Inorganic-Organic Hybrid Talc + Ch

A total of 2.0 g of Talc was added to 7.5 mL of 25% GLA solution and a pre-calculated volume of distilled water. This mixture was subjected to magnetic agitation for 4 h. A total of 2.0 g of Ch was dissolved in 100.0 mL of 1% acetic acid solution and put under magnetic agitation. The pH was adjusted to 5.0 ± 0.2 with a 5.0 mol∙L^−1^ NaOH solution and the agitation was maintained for 4 h [22]. Soon after, the two solutions were mixed under magnetic agitation for 3 h at 30 °C. Posteriorly, system pH was elevated to 11.0 ± 0.5 and the agitation was maintained for 1 h. Finally, the mixture was centrifuged at 3000 rpm for 5 min, washed with alcohol, and oven dried at 60 °C for 24 h. The material was named Talc + Ch and was stored. The synthesis was performed in triplicate.

### 3.5. Characterizations 

#### 3.5.1. Fourier Transform Infrared

Fourier Transform Infrared spectra (FTIR) of the samples were obtained using Vertex 70 spectrometer (Bruker, Billerica, MA, USA), by the 1% KBr pellet method, in the range of 4000 to 400 cm^−1^ wavelengths with 4 cm^−1^ scan resolution and with 64 accumulations.

#### 3.5.2. X-Ray Diffraction

Amiloride, as well as samples before and after adsorption were analyzed by X-ray diffraction (XRD) on a XR-D600A Shimadzu (Nakagyo-ku, Kyoto, Japan), operated at 40 kV and 30 mA, varying 2θ in the range between 5° and 70°. The scanning speed was 2°∙min^−1^, using a step size of 0.02° and a time per step of 0.60 s. A CuKα radiation source was used with a wavelength of 154.06 pm. 

#### 3.5.3. Thermogravimetric Analysis

Thermogravimetric analyses (TG/DTG) were performed on SDT Q600 TA Instruments (TA instrument, New Castle, DE, USA ), made from samples of each material in alumina crucible and using inert atmosphere gas nitrogen (N_2_) flow with a flow rate of 100 mL∙min^−1^, a temperature range from 20 to 1000 °C, and a heating rate of 10 °C∙min^−1^.

### 3.6. Drug Loading Efficiency and Release Tests

#### 3.6.1. Amiloride Loading 

Two samples of each material (Talc, Ch, and Talc + Ch) containing 1.0 g were separately added to 100.0 mL of the 1500 mg∙L^−1^ ALD solution, called Talc/ALD, Ch/ALD, and (Talc + Ch)/ALD, respectively. The systems were mechanically agitated at 160 rpm and 37 °C for 24 h. After this time, the samples were centrifuged, the supernatants were analyzed spectroscopically, and the solids were oven dried at 60 °C for 24 h [70].

The amount of drug incorporated was determined by Equation (3).

(3) $$Q=\;\frac{\left(C_{i}-C_{f}\right)\cdot V}{m}$$

where *Q* is the amount of adsorbed material (mg∙L^−1^), *C_i_* is the initial concentration of adsorbate in the solution (mg∙L^−1^), *C_f_* is the final concentration of adsorbate in solution (mg∙L^−1^), *V* is the volume of solution used in the incorporation process (L), and *m* is the adsorbent mass (g). The percentage of drug release was calculated using Equation (4).

(4) $$ALD\;released\;\left(\%\right)=\;\frac{\left(C_{i}-C_{f}\right)}{C_{i}}\times \;100$$

#### 3.6.2. In Vitro Drug Release Tests 

The release test was performed on a SR-8 Plus Hanson Research, dissolution system using apparatus I (basket), under agitation speed of 50 rpm and 37.0 ± 0.4 °C, with a precisely weighed mass of samples containing the same drug content. A total of 500.0 mL phosphate buffer was used as a dissolution medium (0.1 mol∙L^−1^, pH 7.4), ensuring sink conditions [71,72]. 

Aliquots of 2.0 mL were taken at predetermined time intervals for 4 h, with an immediate replacement of medium at the same temperature. The amount of released ALD was quantified by a UV spectrophotometer. The analyses were performed in triplicate. The mechanisms involved in the drug release process were evaluated through the release of the data obtained, which were treated with different mathematical models (Baker–Lonsdale, Higuchi, Korsmeyer–Peppas, First-Order, Hixson–Crowell, and Weibull).

#### 3.6.3. Statistical Analysis of Data

For determination of significant differences in the drug release kinetics of different formulations, statistical tests including ANOVA and t-test were applied.

## 4. Conclusions

In this work it was possible to obtain synthetic Talc by the sol–gel method and a new organic-inorganic hybrid biomaterial resulting from the crosslinking of Talc and Ch using GLA as crosslinking agent was established. Characterized by XRD, TG/DTG, and FTIR, these materials showed good adsorption capacity of ALD, with the loading of 35.71% for Talc + Ch being better than its precursors Talc and Ch, with respective loading values of 33.56% and 2.74%.

Talc and Talc + Ch materials also showed good results in sustained controlled drug release, where phyllosilicate alone showed a 17% reduction in the amount released at the end of the test, while the hybrid system showed significant release in the first 30 min, then maintained a slow and gradual release. The results suggest the possibility of diffusion rationing to the dissolution medium and use in the modified/controlled release of drugs.

Similar drugs such as triamterene, spironolactone, acetazolamide, bumetanide, furosemide, and piretanide, which also have conjugated π bonds in the aromatic ring in their structure and are capable of promoting hydrogen bonds, probably interact with the proposed systems, similarly to the model drug.

## Figures and Tables

**Table 1:** Adsorbed Mass Amount of Amiloride per Mass of Material in Each System.

Material (1 g)	Adsorbed ALD (mg)
Talc	49.53 ± 0.85
Ch	4.71 ± 0.25
Talc + Ch	55.74 ± 0.93

**Table 2:** Correlation Coefficients of ALD Released in Phosphate Buffer through Different Mathematical Models.

Release Models			Samples	
			Talc/ALD	(Talc + Ch)/ALD
Baker–Lonsdale		*K*	0.004	0.0008
		*r* ^2^	0.8071	0.5813
Higuchi		*K*	4.3281	5.4557
		*r* ^2^	0.7084	0.3564
Korsmeyer–Peppas		*K*	15.7826	27.9740
		*r* ^2^	0.9821	0.9267
		*n*	0.2359	0.1656
First-Order		*K*	0.0054	-
		*r* ^2^	0.2204	0.0000
Hixson–Crowell		*K*	0.0015	-
		*r* ^2^	0.0718	0.0000
Weibull		*K*	60.04	85.51
		*r* ^2^	0.9390	0.9830
		*b*	0.5818	0.1809
